# Needle of Death Thromboelastography Tracings in Severely Bleeding Trauma Patients: A Novel Predictor of Hemorrhagic Blood Failure and Futile Resuscitation?

**DOI:** 10.1111/acem.70192

**Published:** 2025-11-06

**Authors:** Connor M. Bunch, Mark M. Walsh, Ernest E. Moore, Hunter B. Moore, Peter K. Moore, Jeffrey L. Johnson, Samuel J. Thomas, Sarah S. Fox, Daniel F. Lewandowski, Joseph B. Miller

**Affiliations:** ^1^ Departments of Emergency Medicine & Internal Medicine Henry Ford Hospital Detroit Michigan USA; ^2^ Department of Emergency Medicine St. Joseph Regional Medical Center Mishawaka Indiana USA; ^3^ Department of Emergency Medicine Indiana University School of Medicine South Bend Indiana USA; ^4^ Ernest E. Moore Shock Trauma Center Denver Health Denver Colorado USA; ^5^ Department of Surgery University of Colorado Health Science Center Aurora Colorado USA; ^6^ Department of Pulmonary & Critical Care Medicine University of Colorado Anschutz Campus Aurora Colorado USA; ^7^ Department of Trauma & Acute Care Surgery Henry Ford Hospital Detroit Michigan USA; ^8^ Department of Trauma Services Beacon Memorial Hospital South Bend Indiana USA; ^9^ Department of Pulmonary & Critical Care Medicine Henry Ford Hospital Detroit Michigan USA; ^10^ Department of Emergency Medicine Michigan State University Health Sciences East Lansing Michigan USA

Viscoelastic testing (e.g., thromboelastography [TEG]) has been adopted by American and European traumatologists to guide goal‐directed massive transfusion in severely bleeding trauma patients [1, 2]. The increased adoption of viscoelastic testing has been led by trauma surgeons immediately involved in the resuscitation of critically injured patients at level I trauma centers in academic environments. However, many trauma resuscitations in the United States occur in nonacademic centers led only by emergency physicians. It is for this reason that emergency physicians must recognize viscoelastic testing's ability not only to guide blood component resuscitation and predict the need for massive transfusion with the presence of hyperfibrinolysis on initial TEG. Viscoelastic testing, with unique tracing patterns, has also been able to identify terminal coagulopathy on arrival at the emergency room which prognosticates near certain death despite aggressive damage control resuscitation [3, 4]. As Morton et al. aptly posed, we must ask the question for the patient with hemorrhagic blood failure as manifested by these unique TEG tracings, “Are they bleeding because they are dying or dying because they are bleeding?” [5].

Blood product stewardship becomes paramount as the United States' blood supply struggles to recuperate to pre‐COVID pandemic levels. Blood bankers and their suppliers are often left with less than a single day's supply on hand, limiting transfusion to both inpatient essential services and outpatient elective procedures [6]. Severely injured patients who arrive at the emergency room in extremis consume vast quantities of blood products in often futile situations, exhausting local supply in hours' time and hindering equitable distribution of blood products. Additionally, the increased use of fixed ratio 1:1:1 packed red blood cells: fresh frozen plasma: platelets, and the recent promulgation of whole blood trauma resuscitation, depletes blood supplies rapidly [6, 7, 8].

The nascent Suspension of Transfusion and Other Procedures (STOP) criteria proposed six sets of initial clinical (e.g., arrival systolic blood pressure, Glasgow coma scale, and return of spontaneous circulation) and laboratory markers (e.g., fibrinolysis at 30 min [LY30] on TEG and lactate) to predict certain death [7]. Significantly, Van Gent et al. noted the TEG LY30 as the only coagulation laboratory marker with independent predictive value for mortality.

The controversy surrounding the decades‐long search for futility in trauma involves both scientific and ethical challenges, but is fueled primarily by the difficult search for reliable markers that fulfill the criteria of 100% positive predictive value and 100% specificity for certain death [7, 9]. Yet, there is one marker that is highly predictive of futility, and it is the so‐called “death diamond” TEG tracing in patients with hyperfibrinolysis whose tracing is shaped like a diamond due to an LY30 of 100%. The initial report of this tracing in 2015 by Chapman et al. [3] identified a 94% mortality rate for patients with a death diamond with subsequent confirmation of high lethality in 2022 by Farrell et al. [4]. Farrell et al. demonstrated that with serial death diamond tracings with ongoing resuscitation (i.e., two death diamond TEGs despite ongoing massive transfusion and mechanical hemorrhage control), the mortality rate was 100%.

The death diamond tracings identified in the studies by Chapman et al. and Farrell et al. were on the legacy cup‐and‐pin TEG 5000 device, which is being retired. Moreover, the death diamond tracing was seen only on Tissue Factor–enhanced runs, termed the rapid TEG. Therefore, the purpose of this letter is to identify measurements that predict mortality in the newer cartridge‐based TEG 6 s device.

Analysis of the TEG 6 s citrated kaolin assay ([CK], which includes the activator kaolin without the addition of the ex vivo Tissue Factor as in the rapid TEG assay) may allow for more sensitive detection of terminal hemorrhagic blood failure in severely bleeding trauma patients. The original TEG 5000 studies used only the rapid TEG assay which causes the diamond‐shaped tracing by augmenting the extrinsic coagulation pathway with Tissue Factor. To date, no studies analyze the tracing patterns in the kaolin‐only CK assay, a less hypercoagulable and often thought of as a more “pure” clotting test. The reason for the addition of Tissue Factor in the rapid TEG runs is to shorten the time to maximum amplitude, enabling more timely goal‐directed adjudication of platelets and/or fibrinogen concentrates during massive transfusion. The TEG 6 s CK assay demonstrates flatline “needle of death” tracings, an omen observed by clinicians resuscitating these patients which reflects hemostatic failure and usually death. Needle of death tracings have yet to be described in the literature. Therefore, we sought to determine if the CK assay needle of death tracing on the TEG 6 s device is associated with mortality like the rapid TEG death diamond tracing on the TEG 5000 device. We hypothesized that the severely bleeding trauma patient may demonstrate one or both death diamond and needle of death tracings on the TEG 6 s device, and that the presence of either of these tracings holds similar prognostic value along the spectrum of terminal hemorrhagic coagulopathy observed in the dying patient with hemorrhagic blood failure.

This study was approved by the Henry Ford Health Institutional Review Board (No. 17248) under waived consent. We retrospectively identified a convenience sample of 19 TEG 6 s runs from 16 severely bleeding trauma patients who died and whose TEGs conform to the rare death diamond or needle of death tracings. The methodology of the TEG 6 s cartridge system is non‐contact and uses a light‐emitting diode measurement of viscoelastic changes to generate tracings like the cup‐and‐pin technology of the TEG 5000. The TEG 6 s device and cartridges are manufactured by Haemonetics in Braintree, Massachusetts.

As comparisons to the TEG 6 s, this study uses the death diamond definition proposed by Chapman et al. on the rapid TEG 5000 as a time to maximum amplitude < 14 min after initiation and time to total lysis < 30 min after the maximum amplitude [3]. The samples in this study were performed on the TEG 6 s Global Hemostasis with Lysis cartridge which performs both the CK and rapid TEG assays simultaneously (Figure 1).

**FIGURE 1:**
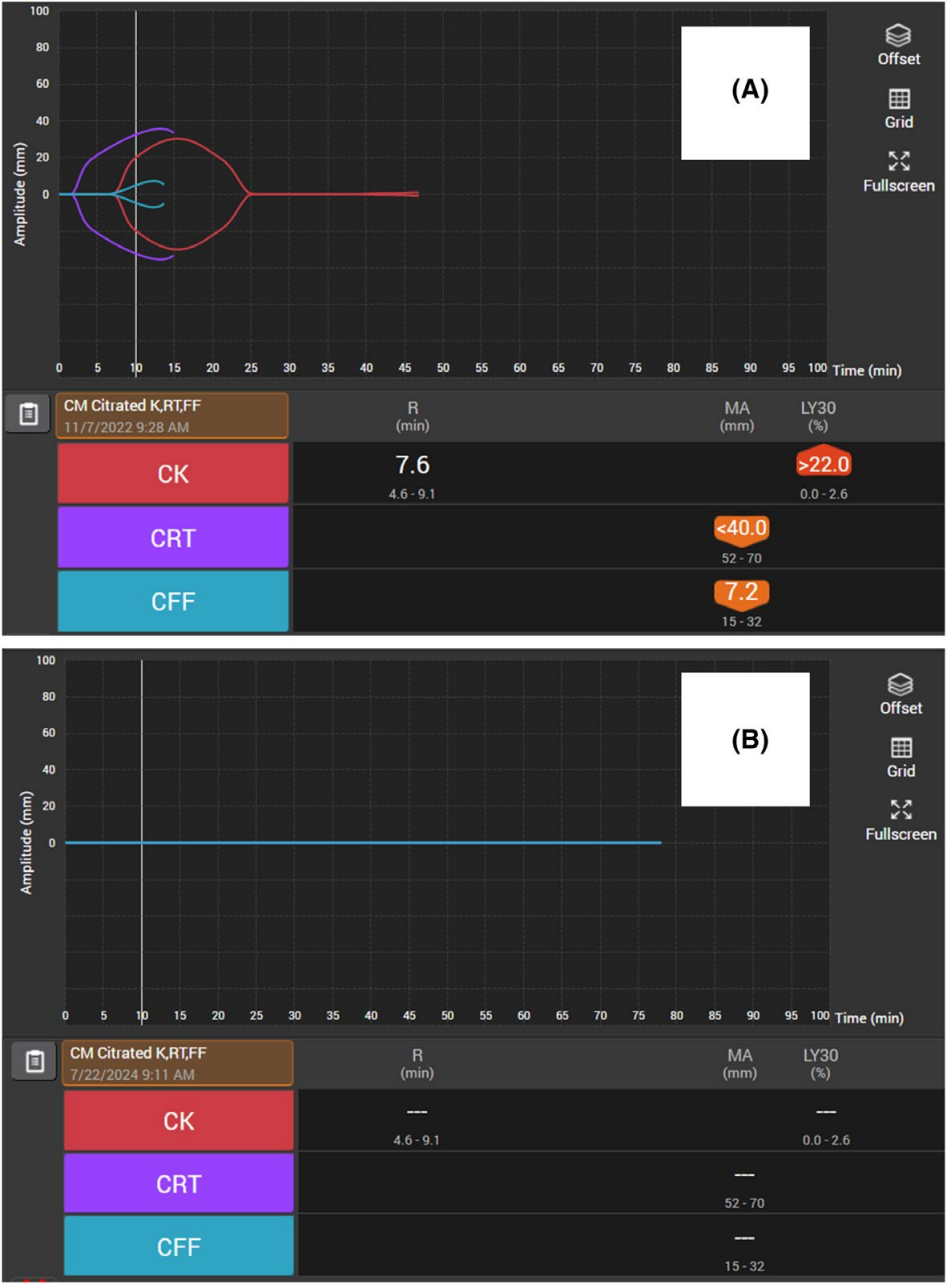
Thromboelastogram (TEG) tracings from two expired, severely injured patients. These tracings were obtained on the cartridge‐based TEG 6 s device. (A) Initial TEG of a 29‐year‐old male who suffered blunt trauma with an injury severity score of 75. This TEG demonstrates the “death diamond” tracing on the citrated kaolin (CK) assay (red tracing), an omen of near certain mortality previously described on the TEG 5000 device. (B) Initial TEG of a 42‐year‐old male who suffered penetrating trauma with an injury severity score of 34. This TEG demonstrates the “needle of death” tracing, which we argue is an equal (if not worse) omen of near certain death manifested by hemorrhagic blood failure. Both patients underwent aggressive damage control resuscitation which was ultimately futile. It is important to note that the death diamond tracing was originally described on the TEG 5000 device rapid TEG assay (which included activators Tissue Factor, kaolin, and calcium chloride) and has now been renamed the citrated rapid TEG (CRT) assay on the TEG 6 s device (the purple tracing in (A), not visible in (B) due to overlapping flatline tracings). The TEG 5000 is being retired and has been supplanted by the multichannel cartridge–based TEG 6 s device. Now, on the TEG 6 s device, the CRT tracing does not report the lysis at 30 min (LY30) value and does not complete the diamond‐shaped tracing due to unspecified manufacturer reasons. Additionally, because the Suspension of Transfusion and Other Procedures (STOP) criteria applies the LY30 of 30%, it will be important for clinicians to visualize TEG 6 s tracings since the maximum reported quantitative value is > 22% LY30. The CK assay, with activators kaolin and calcium chloride, is now the assay which demonstrates the death diamond and needle of death tracings and warrants more validation of its prognostic value.

Review of 19 TEG 6 s tracings from 16 trauma patients who died reveals that the CK may produce parameters that also predict futility like the TEG 5000. Of the 19 CK tracings, 13 were death diamonds with an average time to maximum amplitude of 19.9 ± 8.1 min and time to total lysis of 15.5 ± 7.6 min. Six TEGs included CK needle of death tracings with maximum amplitudes equal to zero. Needle of death and death diamond tracings are associated with death in the TEG 6 s, reflecting previously defined rapid TEG death diamonds as trauma‐associated mortality predictors.

The question “Are they bleeding because they are dying or dying because they are bleeding?” is central to early trauma‐induced coagulopathy, particularly when severe hyperfibrinolysis is present. While hyperfibrinolysis may respond to antifibrinolytics like tranexamic acid and aggressive damage control resuscitation, it often indicates irreversible physiologic collapse and correlates strongly with mortality and massive transfusion needs.

Some view this as an opportunity rather than futility, arguing that markers of extreme physiologic distress, such as the death diamond or needle of death TEG patterns, should prompt aggressive and “heroic” interventions (e.g., resuscitative thoracotomy, cardiopulmonary bypass, aortic balloon occlusion). Though associated with poor outcomes, these TEG phenotypes may justify early, intensive resuscitation rather than therapeutic withdrawal [3].

In resource‐limited settings, however, death diamond pathophysiology might guide retriage decisions, especially when no surgically correctable injuries are present. While not yet a standalone criterion to cease care, it parallels current practice‐limiting invasive efforts to potentially survivable cases such as the now widely accepted limits on emergency room resuscitative thoracotomy [10].

In well‐resourced level I trauma centers, aggressive support is warranted, but if coagulopathy persists (i.e., serial needle of death or death diamond tracings) despite hemorrhage control and transfusion, it may signal irreversible blood failure. Further research is needed to define when hemorrhagic blood failure becomes non‐recoverable and to guide timely, evidence‐based interventions.

The foremost limitation of this study is the convenience nature of the patient population. Additionally, these are rare tracings; many patients who die may not demonstrate hyperfibrinolysis to the degree of causing a death diamond or needle of death tracing. Moreover, we did not analyze all patients with death diamond or needle of death tracings to see if any survived, as we only included those patients who ultimately succumbed to their injuries. Caution must be used in extending these tracing patterns to areas outside of trauma, such as liver transplantation where hyperfibrinolysis is routinely expected in the anhepatic phase of surgery. Moreover, if a death diamond or needle of death tracing is observed in a patient with isolated traumatic brain injury who may serve as an organ donor, this may be another reason to use some resources to extend life temporarily to brain death testing if hemorrhage control may be achieved.

## Author Contributions

Conceptualization: C.M.B., M.M.W., D.F.L., J.B.M. Writing – original draft preparation: C.M.B., M.M.W., J.B.M. Writing – review and editing, Writing – critical revisions: C.M.B., M.M.W., E.E.M., H.B.M., P.K.M., J.L.J., S.J.T., S.S.F., D.F.L., J.B.M. Data collection and analysis: C.M.B., M.M.W., S.J.T., S.S.F., J.B.M. All collaborating authors contributed to the article and approved the submitted version. All authors have read and agreed to the published version of the manuscript.

## Conflicts of Interest

E.E.M. and H.B.M. have received research grants from Haemonetics Corporation outside of the submitted work. The remaining authors declare that the research was conducted in the absence of any commercial or financial relationships that could be construed as potential conflicts of interest.

## Data Availability

The data that support the findings of this study are available from the corresponding author upon reasonable request.
